# Mechanics and Crime

**Published:** 1871-03

**Authors:** 


### MECHANICS AND CRIME.

A BOY under sixteen murdered an old man of eighty-two recently, near New Bedford, Mass. A large number, a very large number, of the criminals in our large cities are youths not out of their teens. In nearly every case it is found that the great cause of this early crime is idleness. Among this army of young criminals are found many of the sons of our hard-working mechanics; of fathers who are supporting their families by day's work. As the family increases, the father has to work the harder, when with approaching age he should have more rest and lighter work; but his sons can't help him, because they do not know how, never having had the chance to learn, not even the trade of their fathers. Twenty years ago this would have been incredible, and there are multitudes now who do not believe the statement, not knowing the fact that there are large societies of mechanics who bind themselves to work for no man who employs over four apprentices; that is, the man who is employed to lay brick, for example, says to his employer, who has ten, twenty, or fifty men working for him, "You shall have but four apprentices." The result is, that among fifty mechanics' families only four boys can have the trade taught them; all others must go at something else. But if other trades do the same thing, there is no place for the children to go to learn a useful and honorable employment; and as activity is a physical necessity for the young, they naturally roam the streets, are attracted to crowds, to the grocery, to the engine-house, to the circus, the theatre, and other disreputable and demoralizing associations, and by rapid and certain downward courses they reach the cock-pits, the dance-house, the jail, the penitentiary, and the gallows. This action of mechanics is not without some show of reason. If a master mason needs twenty men at three dollars a day, and he can get boys apprenticed to him who cost him fifty cents a day, or less, in " victuals and clothes," he clears twenty-five dollars a day, by saving that much in wages, while ten masons have no work to do. Mechanics, families must have bread to eat; and to get that the fathers must have work to do. If they cannot get work the family must starve, or the sons must rob and steal on the street, to make up the deficiency, and the robbing and the stealing end in the prisons or on the gallows.

There is another difficulty. As soon as the apprentice gets a little insight into his trade, he gets tired of working for his " victuals and clothes," wants to earn something for himself, and says, " I might as well get a dollar or two a day as nothing;" thus as soon as he gets to be serviceable to his master, and able to begin to repay him for the trouble of giving him instruction, these half-fledged workmen are glad enough to get employment at half pay; and " setting up shop " for themselves, they are employed by that large class who patronage those who work for the least amount, not discriminating between a piece of work well done and half done. The result is, the price of wages is knocked down, and a man with a family of four or five, to get work at all, must do so at reduced wages, and support that family with the amount that the apprentice workman has for himself alone. The case is a hard one all round. The quickest way of meeting the difficulty is to send children at an early age, say of twelve, or thirteen, to the country to learn farming, or to small towns and villages where trade-unions are not found, to learn useful trades. It might be well to pass a law that no man should collect wages for work done, unless he could show a certificate that he had served out his apprenticeship, signed by his master, on the same principle that clergymen, lawyers, and physicians must show their ability for their callings by examination, diplomas, or otherwise, before they can exercise them.

